# Potential Distribution of Chagas Disease Vectors (Hemiptera, Reduviidae, Triatominae) in Colombia, Based on Ecological Niche Modeling

**DOI:** 10.1155/2016/1439090

**Published:** 2016-12-28

**Authors:** Gabriel Parra-Henao, Laura C. Suárez-Escudero, Sebastián González-Caro

**Affiliations:** ^1^Red Chagas Colombia, Bogotá, Colombia; ^2^Centro de Investigación en Salud para el Trópico (CIST), Universidad Cooperativa de Colombia, Santa Marta, Colombia; ^3^Instituto Colombiano de Medicina Tropical-Universidad CES, Medellín, Colombia; ^4^Servicios Ecosistémicos y Cambio Climático, Jardín Botánico de Medellín, Medellín, Colombia

## Abstract

Ecological niche modeling of Triatominae bugs allow us to establish the local risk of transmission of the parasite* Trypanosoma cruzi, *which causes Chagas disease. This information could help to guide health authority recommendations on infection monitoring, prevention, and control. In this study, we estimated the geographic distribution of triatomine species in Colombia and identified the relationship between landscape structure and climatic factors influencing their occurrence. A total of 2451 records of 4 triatomine species (*Panstrongylus geniculatus*,* Rhodnius pallescens*,* R. prolixus*, and* Triatoma maculata*) were analyzed. The variables that provided more information to explain the ecologic niche of these vectors were related to precipitation, altitude, and temperature. We found that the species with the broadest potential geographic distribution were* P. geniculatus*,* R. pallescens*, and* R. prolixus*. In general, the models predicted the highest occurrence probability of these vectors in the eastern slope of the Eastern Cordillera, the southern region of the Magdalena valley, and the Sierra Nevada of Santa Marta.

## 1. Introduction

Triatomines (Hemiptera, Reduviidae) are hematophagous insects that are considered important vectors of* Trypanosoma cruzi*. They are widely distributed in America with rare occurrences in Eastern Asia, though they are primarily found in the Neotropical region [1]. Disease transmission occurs from southern United States to Argentina, with approximately 25% of the human population at risk [1, 2].

Triatomines have a wide range of climatic and ecological tolerability because they inhabit diverse ecosystems, from humid to dry forests across the Americas [2]. They are found at different altitudes and have high vagility, which allows them to exploit diverse food resources and to find different resting sites, including intra- and peridomiciliary habitats [3–6]. Therefore, at the species level, habitat preferences are influenced by environmental factors, which can strongly drive their distribution [7–9]. Temporal and spatial changes in temperature, precipitation, and humidity affect their biology and ecology, which can alter the risk of transmitting* T. cruzi* [10]. These insects appear to be able to move between microclimates within their habitats while seeking the most favorable conditions [11]. Local environmental determinants can be described as a function of altitude, climate, vegetation type, and land use [11]. For some species of Triatominae, vegetation type appears to be an important predictor of abundance [12]. Likewise, higher Triatominae abundance appears to be associated with perturbed vegetation (agriculture and pasture) [13]. This suggests that deforestation and habitat degradation are important factors contributing to the habitat of some species of triatomine.

Remote sensing, geographic information systems (GIS) and their use for modeling species distribution, have allowed identifying high infestation areas, data simplification, and control efforts. Ecological niche models are empirical or mathematical estimates of species ecological niche in terms of suitable habitat conditions [14, 15]. They permit the association of different ecogeographical variables (e.g., environmental, topographical, and anthropogenic) with the species distribution, by identifying the variables that constrain and define the particular niche. The modeling outcome can be a spatial representation of the favorable habitat for a species occurrence. Therefore, suitability maps for the species, as function of their ecological niche, can be created [16].

Ecological niche models have been used to build potential distribution models for 18 Triatominae species in Brazil [17–19],* Triatoma infestans* in Argentina [12],* T. dimidiata* in México [13], and* Rhodnius pallescens*,* R. prolixus*,* T. dimidiata*, and* Panstrongylus geniculatus* (unpublished data) in Colombia [12, 20–22]. Although there have been some advances in the understanding of the distribution patterns of these vectors, it is necessary to increase our knowledge to refine and improve the use of remotely sensed environmental variables. This will help to describe and predict the distribution of the triatomine species of epidemiological importance because they invade houses and transmit* T. cruzi.*

The Chagas Disease Control Program in Colombia (CDCP) [23] manages vector control programs through entomological surveys in the 20 departments where the disease is endemic. This program allows the identification of transmission risk zones according to entomological indices. However, CDCP does not use spatial technologies like GPS, GIS, and spatial analysis in the endemic departments [24]. The main species transmitting* T. cruzi* in Colombia are* R. prolixus* and* T. dimidiata*, which present a very complex epidemiological cycle that involves domestic, peridomestic, and sylvatic habitats [25, 26]. However, other species such as* P. geniculatus*,* R. pallescens*,* T. maculata*, and* T. venosa* play an important role as secondary vectors of the parasite. These species have greatest association peridomestics and wild habitats and may in the future play an important role in the transmission of the disease. Therefore, monitoring of these species should be included in surveillance programs [25, 26].

In this study, we explore the relationship between the geographical distribution of* P. geniculatus*,* R. pallescens*,* R. prolixus*, and* T. maculata* and different bioclimatic variables, in order to obtain the potential distribution model for each species. We also describe the bioclimatic variables and landscape attributes that best explain the potential distribution of each species in Colombia.

## 2. Material and Methods

### 2.1. Studied Species and Distributional Information

**Figure 1:**
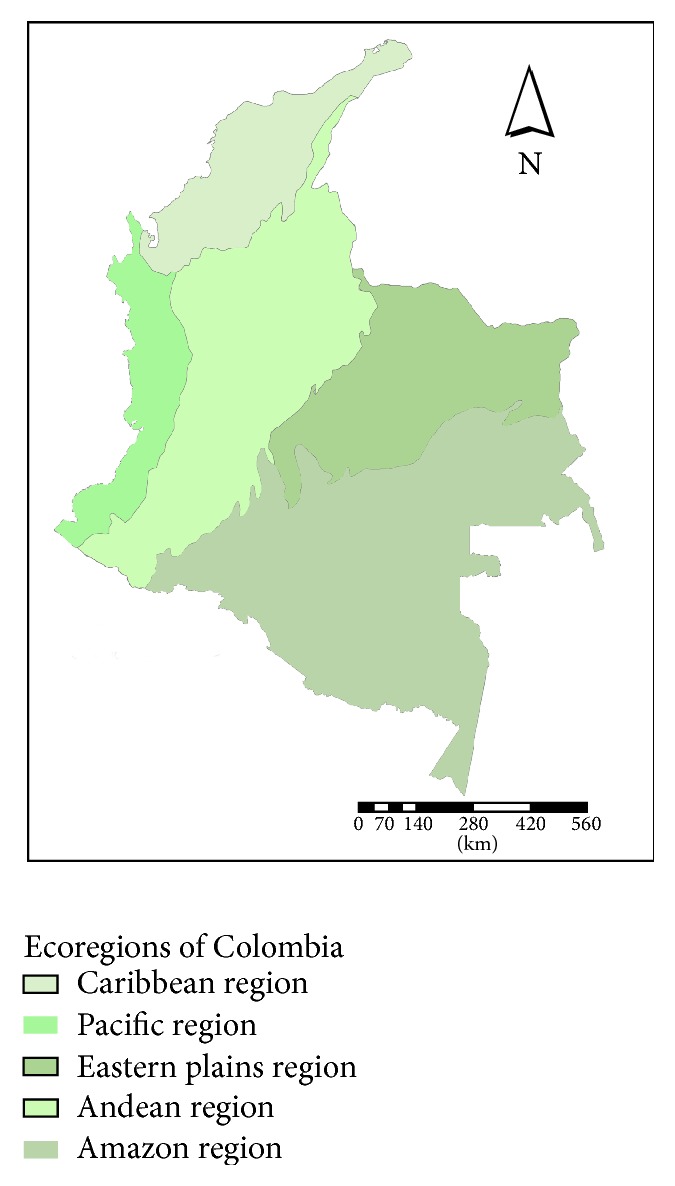
Ecoregions of Colombia.

A database of the occurrence of* Panstrongylus geniculatus*,* Rhodnius pallescens*,* R. prolixus*, and* Triatoma maculata* was obtained for the period from 1998 to 2013 from the Colombian Chagas Network. A total of 5,395 records (2,451 were georeferenced) in 20 departments were obtained:* P. geniculatus *(*n* = 572),* R. pallescens* (*n* = 220),* R. prolixus* (*n* = 1339), and* T. maculata* (*n* = 320). The records were distributed in different ecoregions of the country (Figure 1).

### 2.2. Georeferencing Errors

A descriptive analysis for each georeferenced location was performed in the R project v.3.0.3 [27] with the* sp *package [28] through the “buffer” tool. This analysis described the variation of each bioclimatic variable around a collecting site and allowed us to identify the sensitivity per grid cell to the georeferencing errors. Since climatic-homogeneous areas would not have biases, an influence area of 10 km of radius was generated for each occurrence, and descriptive statistical parameters, such as median and min-max range, were calculated. In addition, linear regression for the occurrence data versus each variable was performed. To avoid influential outliers, we excluded 2.5% of the distribution in each tail from the residual distribution of the linear model. A total of 1281 records were used for the models:* P. geniculatus* (*n* = 422),* R. pallescens* (*n* = 153),* R. prolixus* (*n* = 559), and* T. maculata* (*n* = 147).

### 2.3. Environmental Variables

Nineteen bioclimatic variables were obtained from WorldClim-Global Climate Data, v.1.4 (http://www.worldclim.org/) [29], with a spatial resolution of approximately 1 × 1 km^2^ (30 arc-seconds). In addition, a DEM raster image from WorldClim was also used.

### 2.4. Avoiding Redundancy of Variables

As bioclimatic variables can be correlated with each other [30], a cluster analysis was performed to avoid collinearity and overestimation in the models. The Pearson value threshold was chosen to be *r*_*p*_ < 0.7 (Table 1).

### 2.5. Species Distribution Models and Model Validation

The Maximum Entropy Algorithm, MaxEnt [16], was performed on* dismo *[31] and ENMeval [32] packages in R. ENMeval allow for automatically partition data into evaluation bins. The potential species distribution maps were obtained applying the default parameters and varying the regularization value *β* from 0.25 to 2 (each 0.25), for a total of 8 analyses. We compared models based on Akaike Information Criterion (AIC), where the lower the AIC, the better the model fit [33].

The occurrence data for each species were separated into two sets. 50% of the data points were randomly selected as training points and used for model formulation and selection. The remaining 50% of the records were retained as test points. The MaxEnt model output format was set to logistic. In addition, binary maps were created using the 10th percentile training presence logistic threshold to separate the map into binary predictions. To determine which of the variables contributed more to explain the triatomine distributions, we used MaxEnt's internal Jackknife test of variable importance.

The model's performance was evaluated with the Receiver Operating Characteristic (ROC) curve and the Area Under the curve (AUC), as an estimate of the model performance across these thresholds [30]. A total of eight maps were obtained for each species; to choose the best *β* value we plotted the AUC values versus the *β* value (Table 2) and then the models were run (five replicates) with the regularization value of 0.75, since the models behavior did not change significantly with higher values of *β*.

### 2.6. Landscape Matrix

The suitable zones of potential occurrence were to describe the landscape matrix (mixture of different types of ecosystems) in terms of transformed ecosystems (i.e., due to anthropogenic activities) and natural ecosystems, based on the ecosystems map of Colombia from Etter [34] (Figure 4).

## 3. Results

**Figure 2:**
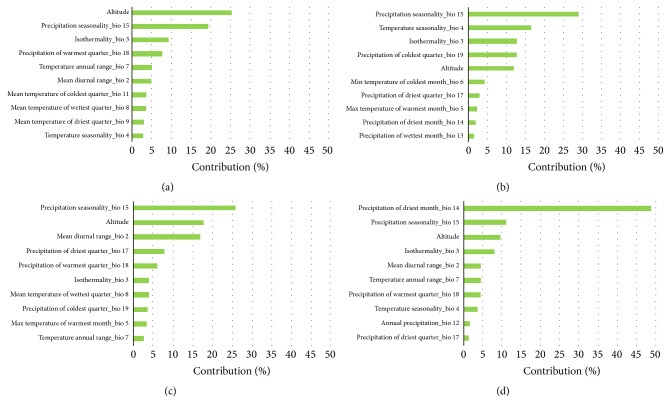
Variation in Triatominae species explained by variation of environmental variables, Method Jackknife: (a)* Panstrongylus geniculatus*; (b)* Rhodnius pallescens*; (c)* Rhodnius prolixus*; (d)* Triatoma maculata.*

The collinearity analysis showed that the variables annual mean temperature (bio 1) and annual precipitation (bio 12) were redundant and precipitation of wettest quarter (bio 16) and only altitude and precipitation seasonality (bio 15) contributed to explain the distribution across the four species (Table 1). The remaining variables contributed differently to each species model (Table 1 and Figure 2). For* P. geniculatus* altitude, precipitation seasonality (bio 15) and isothermality (bio 3) were the variables that most contributed to the model. For* R. pallescens* the three variables that better described the variation in the distribution of the species were precipitation seasonality (bio 15), temperature seasonality (bio 4), and isothermality (bio 3). In the case of* R. prolixus,* precipitation seasonality (bio 15) and altitude and mean diurnal range (bio 2) were the most important variables that explained the model. And for* T. maculata* the variables that better described the distribution were precipitation of driest month (bio 14), precipitation seasonality (bio 15), and altitude.

**Figure 3:**
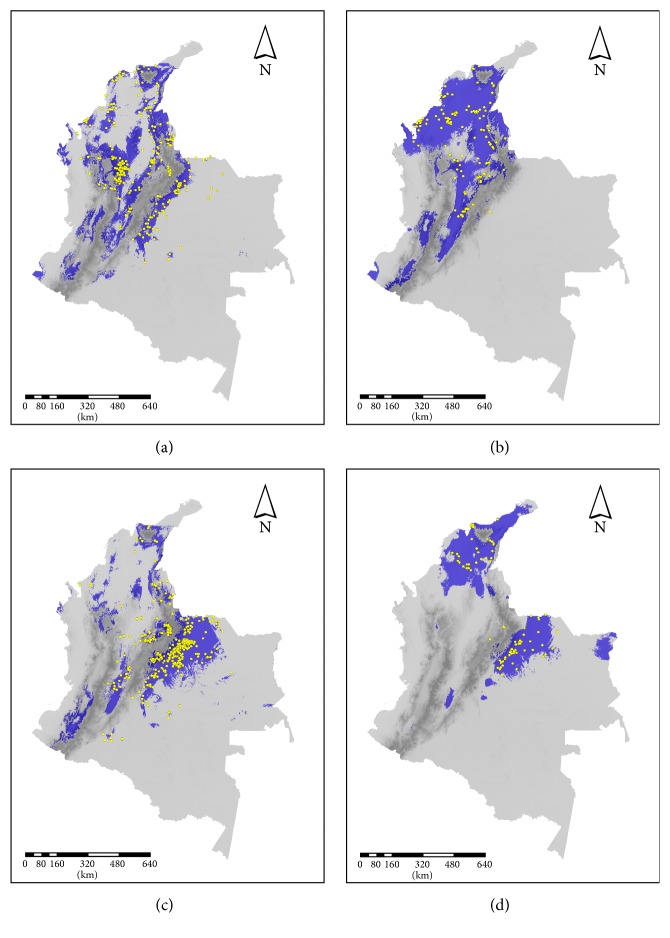
Distributional models of representative Triatominae species from Colombia: (a)* Panstrongylus geniculatus*; (b)* Rhodnius pallescens*; (c)* Rhodnius prolixus*; (d)* Triatoma maculata*. The areas identified as suitable based on climatic information are shown in blue. The yellow points indicate the occurrence data for each species. Dark and light gray represent areas not predicted for the models.

The models predicted the highest occurrence probability in the eastern slopes of Eastern Cordillera, the southern region of the Magdalena valley, and the Sierra Nevada of Santa Marta. In particular,* P. geniculatus *showed a high occurrence probability towards the Sierra Nevada of Santa Marta, north of Central Cordillera to the Serranía de San Lucas, the eastern and western slopes of Eastern Cordillera, and the lowlands of serranía de Perijá (mean AUC = 0.834, sd = 0.012; mean Kappa = 0.737, sd = 0.025). In addition, there are few places with high probability in the southernmost of the Western Cordillera and in the Caribbean coast (Figure 3(a)).* Rhodnius pallescens* showed the widest pattern distribution (mean AUC = 0.906, sd = 0.011; mean Kappa = 0.771, sd = 0.066), with the highest occurrence probability in the Caribbean coast, Magdalena valley, Urabá Gulf, center region of Cauca valley, and lowlands of serranía de Perijá (Figure 3(b)). For* R. prolixus* the occurrence is more limited (mean AUC = 0.881, sd = 0.014; mean Kappa = 0.701, sd = 0.055), with the highest occurrence probability in the eastern slope of Eastern Cordillera towards the Orinoquía region, the southern region of the Magdalena valley, the southern region of Western and Central Cordilleras, and the Sierra Nevada of Santa Martha (Figure 3(c)). Finally,* T. maculata* showed the most restricted pattern (mean AUC = 0.928, sd = 0.003; mean Kappa = 0.791, sd = 0.091), with highest occurrence probability in the Caribbean coast towards the Guajira peninsula, the eastern slope of Eastern Cordillera towards the Orinoquía region, and a restricted region of Orinoquía near the Venezuelan border (Figure 3(d)).

**Figure 4:**
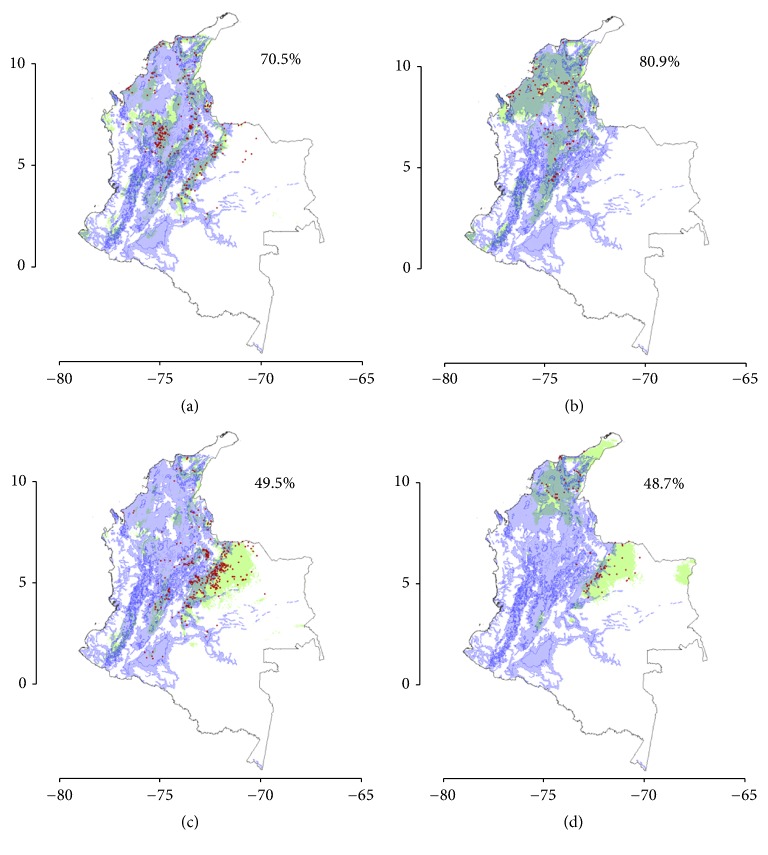
Proportion of species found in transformed ecosystems versus natural ecosystems for the predicted distribution of the Triatominae species. (a)* Panstrongylus geniculatus*; (b)* Rhodnius pallescens*; (c)* Rhodnius prolixus*; (d)* Triatoma maculata*. Green areas show the potential distribution of each of the species, red points are the occurrence data for each species, and blue areas indicate the transformed ecosystem according to the classification of Etter's (1998) ecosystem map. The percentage species distributed in transformed ecosystems is shown in the top right hand corner of each map.

In general, we found that the Triatominae species were more likely to be present in transformed ecosystems (Figure 4(d)):* P. geniculatus *with 70.5%,* R. pallescens *with 80.9%,* R. prolixus *with 49.5%, and finally* T. maculata *with 48.7%.

## 4. Discussion

We found that* P. geniculatus*,* R. pallescens*, and* R. prolixus* have wider distributional ranges than* T. maculata*. This observation is based on the number of ecosystems occupied by the species. Although sampling effort and species knowledge largely influence these results, these findings reveal interesting patterns related to species tolerance to disturbed environments. Human activities that negatively impact the natural ecology of triatomine species, such as deforestation, are likely to encourage adaptation to the domestic environment [35, 36].

The relationship between climatic factors and the geographical distribution of Chagas's disease vectors has been studied by several authors. The studies of Carbajal de la Fuente et al. [18] and Mischler et al. [37] concluded that Triatominae bugs are very sensitive to small variations in temperature and humidity. Carcavallo and Barreto [38] classified the diversity of the Triatominae species in two well-defined seasons with a dry period and a rainy period. The same author associated high population densities with long seasons of dryness and high temperatures. Zeledón and Rabinovich [4] analyzed the influence of climatic factors on different Triatominae species, such as* T. infestans*,* R. prolixus*, and* P. megistus.* They concluded that the most important climatic determinants of the geographical distribution of these vectors were temperature and relative humidity.

We found that variations in the predicted distribution of* P. geniculatus* were primarily explained by altitude, precipitation seasonality (bio 15), and isothermality (bio 3). A large proportion of the potential range of this species is associated with transformed ecosystems. In Colombia, according to Guhl et al. [25]* P. geniculatus* has been reported in 25 departments. The main habitats are burrows and nesting places of marsupials, bats, rodents, and birds, but adult specimens have also been collected from peridomiciles and houses, as these bugs are strongly attracted by artificial lights [39]. This species has been found at altitudes close to 1700 m.a.s.l. (metres above sea level) [40]. Subsequently, it has been speculated that, after an initial adaptation to environments of high humidity in the Amazon, its association with the humid microclimate of armadillo burrows has facilitated its broad geographical distribution beyond the limits of the Amazon [35]. A few other species are also widely distributed, possibly due to their potential to exploit a broader range of habitats or because their ecological niches are widespread. This species is more sensitive to environmental devastation due to its microclimatic requirements. Therefore, in areas of rapid and intense deforestation, the species is less likely to survive compared to where the changes occur on a smaller scale or more slowly [41].

*Rhodnius pallescens* has been reported in Belize, Nicaragua, Costa Rica, Panama, and Colombia, where it inhabits sylvatic environments and it is often found in human dwellings, although without intradomestic colonies [20]. Colonies of the species are also found in sylvatic ecotopes such as crowns of at least four species of palms:* Attalea butyracea, Cocos nucifera, Elaeis oleífera*, and* Oenocarpus bataua. *[42, 43]. The palm tree* A. butyracea* is its primary biotope. These palms, besides serving as shelter for triatomines, are occasional habitats for a great diversity of fauna of mammals, which in turn are a food source for triatomine bugs and reservoirs of the parasite. During the dry season, palms offer optimum humidity and temperature conditions for the development and multiplication of both triatomine and its associated fauna. The leaves are used for thatching houses, a situation that helps create foci of infestation because it promotes the transfer of the Triatominae to the house. The wide potential distribution of* R. pallescens* can be explained by the distribution of the four species of palms mentioned above. This species has a wide geographical distribution in the country and is considered a potential problem since it is a candidate to replace the domestic* R. prolixus* when the latter will be eliminated from houses as a consequence of control campaigns [42]. Our model for* R. pallescens *coincided with previous models for the species [44]. For* R. pallescens* the predicted areas in the Caribbean plains have the ecosystems of tropical rain forest and tropical dry forests.

*Rhodnius prolixus* has a restricted geographical potential distribution, confined to the Andean region. Possibly, its spatial range has decreased due the control actions carried out by the secretaries of health of the departments of Boyacá, Cundinamarca, and Santander. However, the obtained predicted distribution agrees with the areas that have historically been most prevalent for Chagas disease in the country. We report new areas of potential distribution of* R. prolixus* to the Oriental Plains that coincide with industrial plantations of oil palm (*Elaeis guineensis*). Sylvatic* R. prolixus* have been reported in these palms [45]. Also we observed a potential distribution to the south of the country which should be studied in more detail.

Our findings coincide in part with previous distributional models generated for* R. prolixus* in Colombia. These models showed two distinct distributional areas [21]: one geographical zone at the east of Eastern Cordillera, associated with the environmental variables used in the work of Guhl [21], normalized difference vegetation index (NDVI), land surface temperature (LST), middle infrared (MIR), air temperature (TEMP), and altitude, and another zone at Northwestern of the Central Cordillera with low association with these variables. Guhl [21] found little association between environmental variables, such as temperature, altitude, and vegetation cover, northwest of the Central Cordillera. The lack of association was attributed to the passive dispersion of vector populations by human migration. In contrast, other studies have shown a high association between relative humidity, maximum temperature, and species presence [11].* Rhodnius prolixus* is the most efficient vector of* T. cruzi*, due to its biological features, namely, fast reproduction rates, adaptation to the human environment, and high rates of defecation and infection [25]. In this study, we found that this species is potentially distributed across highly populated regions of the country (towards the northern portion of the Eastern Cordillera in Santander and Boyacá departments; Figure 3(c)). Furthermore, it is predicted to be present in the Oriental Plains region (Casanare and Meta departments). These findings are in agreement with the known distribution of* R. prolixus *in Colombia.

This study is among the first ones that has attempted [20–22] to model the distribution of* T. maculata *in Colombia. The distribution of this species seems to be mainly explained by precipitation variables (Figure 2(d)). Because the highest occurrence probability was towards the Caribbean coast and Oriental Plains, and the variation of seasonality precipitation would not affect the presence of the species, and* T. maculata* may be considered an emerging vector in the Northern Andean countries (Venezuela and Colombia). In some areas of Venezuela and Colombia, this species has the capacity to colonize human dwellings and may be responsible for Chagas disease transmission [25]. Usually, this species is peridomestic [46], found in outer walls of the houses, chicken coops, dove nests, and sylvatic environments: dead trunks, palm trees* Attalea* complex, bromeliads, caves, and birds' nests [47].* Triatoma maculata* can adapt quickly to stable artificial ecotopes when their natural habitats are destroyed. It survives in very dry regions, such as Margarita Island and Paraguaná Peninsula [8]. Wild and peridomestic* T. maculata* is also found in Brazil (Roraima state), Guiana, French Guiana, and Suriname. This species has distribution only in the sylvatic environment [48].* Triatoma maculata* also occurs in Aruba, Bonaire, Curacao, Surinam, Guyana, and Brazil (Roraima) [49, 50]. In Colombia, it is reported in the departments of Atlántico, Bolívar, Boyacá, Casanare, Cesar, La Guajira, Magdalena, Meta, Santander, and Vichada [51–54].

We found a high coincidence of the predicted distribution of* T. maculata* with transformed and environmentally degraded ecosystems, which in these areas coincide with the domiciliary and peridomiciliary habitats.

As it has been suggested for some Triatominae species (e.g., Romaña et al.) [55], other sources of ecological information, such as the distribution of reservoirs (e.g., American marsupials of the family Didelphidae) and vegetation type (e.g., palms like* Attalea butyracea* (Mutis ex L. f.) Wess. Boer), should be considered and included within the models. Further, validation of results via field investigations to identify present species should be conducted.

**Figure 5:**
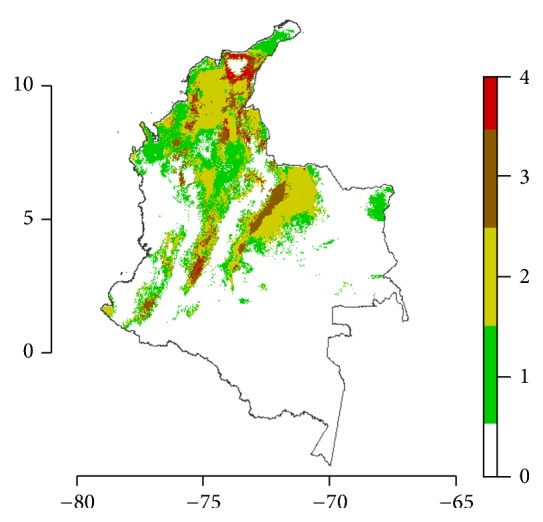
Combined diversity map for Triatominae species in Colombia. The color scale shows the following: white, zero species; green, one species; light green, two species; brown, three species; and red, four species.

According to the potential distribution of each species, we defined potential sympatric areas in the country, as an attempt to identify areas where health authorities have to target and improve the entomological surveillance of triatomines.  Figure 5 shows that the Sierra Nevada of Santa Marta, the Caribbean coast, Catatumbo, and the southern region of Magdalena valley are areas at the confluence of the four species studied (red color).

The lack of species towards the east of the country could be due to the absence or scarcity of records in this area. Even though this zone of Colombia is the least populated area in the country, entomological surveys in these regions could improve our knowledge of the real vector distribution and consequently the transmission risk of Chagas disease.

## 5. Conclusion

This study highlights the relationship between environmental factors and* P. geniculatus, R. pallescens, R. prolixus*, and* T. maculata* in Colombia and the importance of GIS and modeling tools to improve mapping efforts. These tools should form part of the official prevention and control plans for vector borne diseases. Previously several aspects of the ecoepidemiology of Triatominae species were unknown and this study helps to identify potential geographic regions where these species can thrive. Knowledge of the biology of vectors is central to choose complementary measures to traditional vector control and surveillance strategies. Ultimately, this information contributes to the understanding of the dynamics of transmission of* T. cruzi* at local, regional, and national levels.

## Figures and Tables

**Table 1:** Uncorrelated (*r*_*p*_ < 0.7) environmental variables used in the species distribution models for the triatomine species.

Variable	Description	*Panstrongylus geniculatus*	*Rhodnius pallescens*	*Rhodnius prolixus*	*Triatoma maculata*
—	Altitude	X	X	X	X
Bio 2	Mean diurnal range	X	—	X	—
Bio 3	Isothermality	—	X	X	X
Bio 4	Temperature seasonality	—	X	—	—
Bio 5	Max temperature of warmest month	X	X	—	—
Bio 6	Min temperature of coldest month	X	—	—	—
Bio 7	Temperature annual range	X	—	—	—
Bio 8	Mean temperature of wettest quarter	—	X	X	—
Bio 9	Mean temperature of driest quarter	X	—	—	—
Bio 10	Mean temperature of warmest quarter	X	—	—	—
Bio 11	Mean temperature of coldest quarter	—	—	—	X
Bio 13	Precipitation of wettest month	X	—	—	—
Bio 14	Precipitation of driest month	—	—	—	X
Bio 15	Precipitation seasonality	X	X	X	X
Bio 17	Precipitation of driest quarter	—	—	X	—
Bio 18	Precipitation of warmest quarter	—	—	X	X
Bio 19	Precipitation of coldest quarter	—	X	X	X

**Table 2:** AUC mean values calculated for each map with varying regularization *β* parameter.

Species	*β*							
	0.25	0.5	0.75	1	1.25	1.5	1.75	2
*Panstrongylus geniculatus*	0.7871	0.9268	0.9399	0.9357	0.9401	0.9373	0.9346	0.9329
*Rhodnius pallescens*	0.9852	0.9805	0.9755	0.968	0.9612	0.9587	0.9567	0.9552
*Rhodnius prolixus*	0.9617	0.9616	0.9634	0.9622	0.9621	0.9591	0.9584	0.9578
*Triatoma maculata*	0.8264	0.9903	0.9891	0.991	0.9913	0.9915	0.9913	0.9907

## References

[1] Schofield C. J., Galvão C. (2009). Classification, evolution, and species groups within the Triatominae. *Acta Tropica*.

[2] Zeledón R., Montenegro V. M., Zeledón O. (2001). Evidence of colonization of man-made ecotopes by *Triatoma dimidiata* (Latreille, 1811) in Costa Rica. *Memórias do Instituto Oswaldo Cruz*.

[3] Arzube M. (1966). Investigación de la fuente alimenticia de *Triatoma dimidiata* Latreille, 1811 (Hemiptera: Reduviidae) mediante la reacción de precipitina. *Revista Ecuatoriana de Higiene y Medicina Tropical*.

[4] Zeledón R., Rabinovich J. E. (1981). Chagas disease: an ecological appraisal with special emphasis on its insect vectors. *Annual Review of Entomology*.

[5] Tabaru Y., Monroy C., Rodas A., Mejia M., Rosales R. (1999). The geographical distribution of vectors of Chagas' disease and populations at risk of infection in Guatemala. *Medical Entomology and Zoology*.

[6] Sasaki H., Rosales R., Tabaru Y. (2003). Host feeding profiles of *Rhodnius prolixus* and *Triatoma dimidiata* in Guatemala (Hemiptera: Reduviidae: Triatominae). *Zoology*.

[7] Galvão C., Jurberg J., Carcavallo R. U. (1998). Distribuição geográfica e dispersão alti-latitudinal de alguns gêneros e espécies da tribo triatomini jeannel. *Memórias do Instituto Oswaldo Cruz*.

[8] Carcavallo R. U. (1999). Climatic factors related to Chagas disease transmission. *Memórias do Instituto Oswaldo Cruz*.

[9] Ramsey J. M., Ordoñez R., Cruz-Celis A. (2000). Distribution of domestic triatominae and stratification of Chagas disease transmission in Oaxaca, Mexico. *Medical and Veterinary Entomology*.

[10] Githeko A. K., Lindsay S. W., Confalonieri U. E., Patz J. A. (2000). Climate change and vector-borne diseases: a regional analysis. *Bulletin of the World Health Organization*.

[11] Bustamante D. M., Monroy M. C., Rodas A. G., Juarez J. A., Malone J. B. (2007). Environmental determinants of the distribution of Chagas disease vectors in south-eastern Guatemala. *Geospatial Health*.

[12] Gorla D. E. (2002). Variables ambientales registradas por sensores remotos como indicadores de la distribución geográfica de *Triatoma infestans* (Heteroptera: Reduviidae). *Austral Ecology*.

[13] Dumonteil E., Gourbière S. (2004). Predicting *Triatoma dimidiata* abundance and infection rate: a risk map for natural transmission of Chagas disease in the Yucatán Peninsula of Mexico. *The American Journal of Tropical Medicine and Hygiene*.

[14] Guisan A., Zimmermann N. E. (2000). Predictive habitat distribution models in ecology. *Ecological Modelling*.

[15] Morin X., Lechowicz M. J. (2008). Contemporary perspectives on the niche that can improve models of species range shifts under climate change. *Biology Letters*.

[16] Phillips S. J., Anderson R. P., Schapire R. E. (2006). Maximum entropy modeling of species geographic distributions. *Ecological Modelling*.

[17] Costa J., Peterson A. T., Beard C. B. (2002). Ecologic niche modeling and differentiation of populations of *Triatoma brasiliensis* neiva, 1911, the most important Chagas' disease vector in Northeastern Brazil (hemiptera, reduviidae, triatominae). *The American Journal of Tropical Medicine and Hygiene*.

[18] Carbajal de la Fuente A. L., Porcasi X., Noireau F., Diotaiuti L., Gorla D. E. (2009). The association between the geographic distribution of *Triatoma pseudomaculata* and *Triatoma wygodzinskyi* (Hemiptera: Reduviidae) with environmental variables recorded by remote sensors. *Infection, Genetics and Evolution*.

[19] Gurgel-Gonçalves R., Galvão C., Costa J., Peterson A. T. (2012). Geographic distribution of Chagas disease vectors in Brazil based on ecological niche modeling. *Journal of Tropical Medicine*.

[20] Arboleda S., Gorla D. E., Porcasi X., Saldaña A., Calzada J., Jaramillo-O N. (2009). Development of a geographical distribution model of *Rhodnius pallescens* Barber, 1932 using environmental data recorded by remote sensing. *Infection, Genetics and Evolution*.

[21] Ghul F. (2010). Variables ambientales, sensores remotos y sistemas de información geográfica aplicados al estudio de la distribución de *Rhodnius prolixu*s en Colombia. *Revista de la Academia Colombiana de Ciencias*.

[22] Parra-Henao G., Segura A., Jaramillo N. (2011). Use of geographical information systems and remote sensing tools for the generation of predictive models of *Triatoma dimidiata* (Latreille, 1811) distribution in Colombia. *Biomédica*.

[23] Minesterio de la Protección Social (2010). *Guía Protocolo para la vigilancia en Salud Pública de Chagas*.

[24] World Health Organization (2012). *Control of Chagas Disease. Second Report of the WHO Expert Committee*.

[25] Guhl F., Aguilera G., Pinto N., Vergara D. (2007). Updated geographical distribution and ecoepidemiology of the triatomine fauna (Reduviidae: Triatominae) in Colombia. *Biomedica*.

[26] Parra-Henao G., Cardona Á. S., Quirós-Gómez O., Angulo V., Alexander N. (2015). House-level risk factors for *Triatoma dimidiata* infestation in Colombia. *American Journal of Tropical Medicine and Hygiene*.

[27] Core Team R. (2014). *R: A Language and Environment for Statistical*.

[28] Pebesma E. J., Bivand R. S. (2005). Classes and methods for spatial data in R. *R News*.

[29] Hijmans R. J., Cameron S. E., Parra J. L., Jones P. G., Jarvis A. (2005). Very high resolution interpolated climate surfaces for global land areas. *International Journal of Climatology*.

[30] Pereira J. M., Almeida P. S. D., Sousa A. V. D., Paula A. M. D., Machado R. B., Gurgel-Gonçalves R. (2013). Climatic factors influencing triatomine occurrence in Central-West Brazil. *Memórias do Instituto Oswaldo Cruz*.

[31] Hijmans R. J., Phillips S., Leathwick J.

[32] Muscarella R., Galante P. J., Soley-Guardia M. (2014). ENMeval: an R package for conducting spatially independent evaluations and estimating optimal model complexity for Maxent ecological niche models. *Methods in Ecology and Evolution*.

[33] Lowe R., Bailey T. C., Stephenson D. B. (2011). Spatio-temporal modelling of climate-sensitive disease risk: towards an early warning system for dengue in Brazil. *Computers & Geosciences*.

[34] Etter A. (1998). *Mapa General de Ecosistemas de Colombia (Escala 1: 2,000.000)*.

[35] Abad-Franch F., Monteiro F. A. (2007). Biogeography and evolution of Amazonian triatomines (Heteroptera: Reduviidae): implications for Chagas disease surveillance in humid forest ecoregions. *Memórias do Instituto Oswaldo Cruz*.

[36] Abad-Franch F., Monteiro F. A., Jaramillo O. N., Gurgel-Gonçalves R., Dias F. B. S., Diotaiuti L. (2009). Ecology, evolution, and the long-term surveillance of vector-borne Chagas disease: a multi-scale appraisal of the tribe Rhodniini (Triatominae). *Acta Tropica*.

[37] Mischler P., Kearney M., Mccarroll J. C., Scholte R. G. C., Vounatsou P., Malone J. B. (2012). Environmental and socio-economic risk modelling for Chagas disease in Bolivia. *Geospatial Health*.

[38] Carcavallo R. U., Barreto P. (1976). Una nueva especie de *Rhodnius* Stål (Hemiptera, Reduviidae, Triatominae) de Colombia. *Boletín de la Dirección de Malariología y Saneamiento Ambiental*.

[39] Curto de Casas S. I., Casas R. U., Mena Segura C. A. (1994). Bioclimatic factors of Triatominae distribution: useful techniques for studies on climate change. *Entomologia y Vectores*.

[40] Curto de S. I., Casas R. U., Galíndez-Girón I I. (1996). Geographical distribution and alti-latitudinal dispersion of species of *Panstrongylus* (Hemiptera, Reduviidae, Triatominae, Triatomini). *Entomologia y Vectores*.

[41] Vallejo G. A. Grupo de trabajo sobre biología, evolución y comportamiento de Triatominae en hábitats selváticos.

[42] Jaramillo N., Schofield C. J., Gorla D. (2000). The role of *Rhodnius pallescens* as a vector of Chagas disease in Colombia and Panama. *Research and Reviews in Parasitology*.

[43] Moreno J., Jaramillo N. Estudios epidemiológicos sobre la enfermedad de Chagas en los Departamentos de Antioquia, Sucre y Tolima, Colombia.

[44] Arboleda S., Triana-Chávez O., Mejía-Jaramillo A. M., Gómez-Palacio A. M. (2011). Aplicación de los sistemas de información geográfica (SIG) en el estudio de la distribución de los vectores de la enfermedad de Chagas. *Fronteras de Investigación de Investigación en Enfermedades Infecciosas: Modelo Enfermedad de Chagas*.

[45] Abad-Franch F., Pavan M. G., Jaramillo-O N. (2013). *Rhodnius barretti*, a new species of Triatominae (Hemiptera: Reduviidae) from western Amazonia. *Memorias do Instituto Oswaldo Cruz*.

[46] Corredor A., Santacruz M. M., Paez S. (1990). *Distribución de los Triatominos Domiciliarios en Colombia*.

[47] Briceño Z. M., Gil A., Giménez E. Z. (2006). *Importancia de Triatoma maculata y Pastrongylus geniculatus en la transmisión de la enfermedad de Chagas en el Estado Lara*.

[48] Galvão C., Carcavallo R., Rocha D. D., Jurberg J. (2003). A checklist of the current valid species of the subfamily Triatominae Jeannel, 1919 (Hemiptera, Reduviidae) and their geographical distribution, with nomenclatural and taxonomic notes. *Zootaxa*.

[49] Lent H., Wygodzinsky P. (1979). Revision of the Triatominae (Hemiptera, Reduviidae), and their significance as vectors of Chagas' disease. *Bulletin of the American Museum of Natural History*.

[50] Dujardin J. P., Schofield C. J., Panzera F. (2002). *Los Vectores de la Enfermedad de Chagas*.

[51] Cortés L. A., Suárez H. A. (2005). Triatominos (Reduviidae: Triatominae) en un foco de enfermedad de Chagas en Talaigua Nuevo (Bolívar, Colombia). *Biomédica*.

[52] Molina J. A., Gualdrón L. E., Brochero H. L., Olano V. A., Barrios D., Guhl F. (2000). Distribución actual e importancia epidemiológica de las especies de triatominos (Reduviidae: Triatominae) en Colombia. *Biomédica*.

[53] Gómez-Melendro E. N., Hernández C., González-Uribe C., Brochero H. (2014). First record of Triatoma maculata (Erichson, 1848) (Hemiptera: Reduviidae: Triatomini) in the municipality of Riohacha, La Guajira-Colombia. *Frontiers in Public Health*.

[54] Cantillo-Barraza O., Gómez-Palacio A., Salazar D. (2010). Distribución geográfica y ecoepidemiología de la fauna de triatominos (Reduviidae: Triatominae) en la Isla Margarita del departamento de Bolívar, Colombia. *Biomédica*.

[55] Romaña C. A., Pizarro J. C., Rodas E., Guilbert E. (1999). Palm trees as ecological indicators of risk areas for Chagas disease. *Transactions of the Royal Society of Tropical Medicine and Hygiene*.

